# Gait Feature Analysis and Recognition Under Coupled Weight Support and Bearing Conditions via a Domain-Adaptive Gradient Boosting Machine

**DOI:** 10.3390/bios16100585

**Published:** 2026-10-19

**Authors:** Junyao Wang, Qinglin Jia, Xinqiang Zhu

**Affiliations:** 1 Flight College, Shandong University of Aeronautics, Binzhou 256600, China; 15244527307@163.com (Q.J.); 19558903553@163.com (X.Z.); 2 School of Mechanical Engineer, University of Science and Technology Beijing, Beijing 100083, China

**Keywords:** body weight support, weight bearing, gait feature, plantar pressure, domain-adaptive gradient boosting machine

## Abstract

Accurate recognition of gait characteristics under coupled body weight support and weight-bearing conditions is the core foundation for gravity adaptation strategy development.To solve this problem, this paper proposed a domain-adaptive gradient boosting machine (DAGBM) algorithm. The DAGBM algorithm added a cross-individual domain-adaptive regularization module to the standard GBM framework. It minimized both classification loss and maximum mean discrepancy (MMD) to align feature spaces across different subjects. It also worked with an individual–environment feature decoupling model to further improve generalization. Comparative experiments were conducted on a three-point suspension low-gravity test bed. A plantar pressure sensor with 48 points was used to collect gait data. This paper also extracted peak pressure, pressure–time integral, regional pressure ratio, and whole-foot center of pressure (CoP) trajectory standard deviation as features. DAGBM achieves 96.8% accuracy for seven body weight support levels, 97.4% accuracy for four weight-bearing levels, and 94.3% accuracy for all 28 fine-grained coupled conditions. DAGBM significantly outperforms both SGBoost and XGBoost algorithms, which exhibit favorable robustness under varied mechanical loading conditions and serve as a methodological reference for gait recognition research under aerospace-mimicking mechanical conditions.

## 1. Introduction

Human gait is a periodic motor behavior synergistically regulated by the central nervous system and the musculoskeletal system. Its biomechanical characteristics can accurately reflect human motor control ability and physiological functional status [1]. Under the dual coupling of partial body weight support (PBWS) and weight bearing, PBWS is also defined as gravity unloading, which refers to a technical means that offsets a preset proportion of human body weight by means of suspension support, buoyancy effect, or mechanical auxiliary intervention [2]. The detection and recognition of human gait possess essential theoretical significance and practical engineering value in aerospace engineering and military training, and have emerged as a core research focus in the above two critical disciplines. In aerospace engineering, PBWS load-coupled gait training is a key method to improve muscle atrophy and bone loss in astronauts under long-term orbital low gravity [3]. Gait regulation rules directly govern training scheme optimization and protective effects. In military training, gait stability and motor economy under coupled gravitational and carried load conditions are key determinants of combat performance and injury prevention during field missions [4]. In summary, investigations into multi-condition coupled gait can elucidate the regulatory mechanisms of human gait under complex loading environments and refine the theoretical framework of sports biomechanics. They also provide scientific guidance for the development of training protocols and the optimization of protective strategies, supply robust data support for a wide range of application scenarios, and thus hold substantial practical value.

In recent years, flexible thin-film pressure sensors have enabled dynamic and continuous monitoring of the full plantar pressure distribution due to their advantages of high flexibility, high sensitivity, fast dynamic response, and strong wearability [5,6]. They have become a core technical approach for plantar pressure data collection under complex working conditions. Based on wearable plantar pressure sensing technology, scholars worldwide have conducted extensive research on gait feature analysis and recognition under various conditions. He et al. used a PBWS harness and force plate to analyze plantar pressure distribution characteristics during level walking under partial weight reduction conditions and found that PBWS significantly reduced peak plantar pressure [7], but did not conduct in-depth investigations on multi-level weight reduction training. Kumar et al. adopted interventions such as PBWS and resistance exercise for musculoskeletal protection of astronauts, aiming to reduce the risk of orthopedic injuries induced by microgravity and maintain on-orbit physiological health [8]. However, such strategies lack in-flight load assessment capacity and rely heavily on manual cross-domain analysis. Consequently, they struggle to meet the demands for precise protection of astronauts’ musculoskeletal health in long-duration deep-space mission scenarios.

Existing studies are predominantly limited to isolated single-factor conditions: either standalone low-gravity/PBWS environments or constant load scenarios. Few works have systematically investigated gait characteristics under the full gradient of PBWS-load dual-coupling conditions [9,10]. Two core scientific and technical gaps still exist: firstly, the coverage of working conditions is incomplete. Existing coupled condition studies mostly focus on a narrow range of gravity unloading levels and lack full factor-coupled experimental designs covering full gradients of PBWS and load carriage; secondly, the cross-individual generalization ability of existing gait recognition models is insufficient. Most models ignore the interference of individual biomechanical differences on environment-sensitive features, struggle to eliminate feature redundancy caused by individual variations, and fail to achieve high-precision, high-stability recognition among different individuals under complex coupled conditions.

To address the above research gaps, this paper takes human gait under partial body weight support and weight-bearing conditions as the research object, and designs a gait experiment covering gravity unloading levels of 0–60% and load levels of 0–30% body weight. A 48-channel flexible thin-film plantar pressure sensor is used to accurately collect plantar pressure data under various conditions. A multi-dimensional gait feature extraction method based on plantar region aggregation is proposed. An individual–environment feature decoupling model is constructed to eliminate the interference of individual biomechanical differences and screen robust features highly sensitive to coupled working conditions. A gait condition recognition model is established based on the DAGBM algorithm, achieving high-precision classification and recognition of gravity unloading levels, load ratio levels, and their coupled conditions, with performance compared against traditional pattern recognition algorithms. The results provide a feasible technical approach for astronaut walking state monitoring, load evaluation, and gravity adaptation strategy research in deep-space exploration.

The remainder of this paper is structured as follows. Section 2 illustrates the overall research framework of this paper, followed by the dynamic analysis of gait under partial body weight support conditions, mechanical derivation of the three-point suspension unloading system, and decoupling modeling for coupled gait characteristics, as well as the establishment of the domain-adaptive gradient algorithm model. Section 3 details the overall experimental design, data acquisition workflow, and extraction approaches for multi-dimensional plantar pressure gait features. Section 4 quantitatively investigates the variational laws of typical gait characteristic parameters under varying body weight support and plantar weight-bearing coupling conditions. Section 5 implements comparative evaluations of the proposed adaptive gradient algorithm against alternative recognition methods, including single-condition classification, coupled-condition identification, and cross-subject generalization performance validation. Finally, in Section 6, we present conclusions and propose potential orientations for subsequent follow-up research.

## 2. Methods

### 2.1. Overall Research Framework

**Figure 1:**
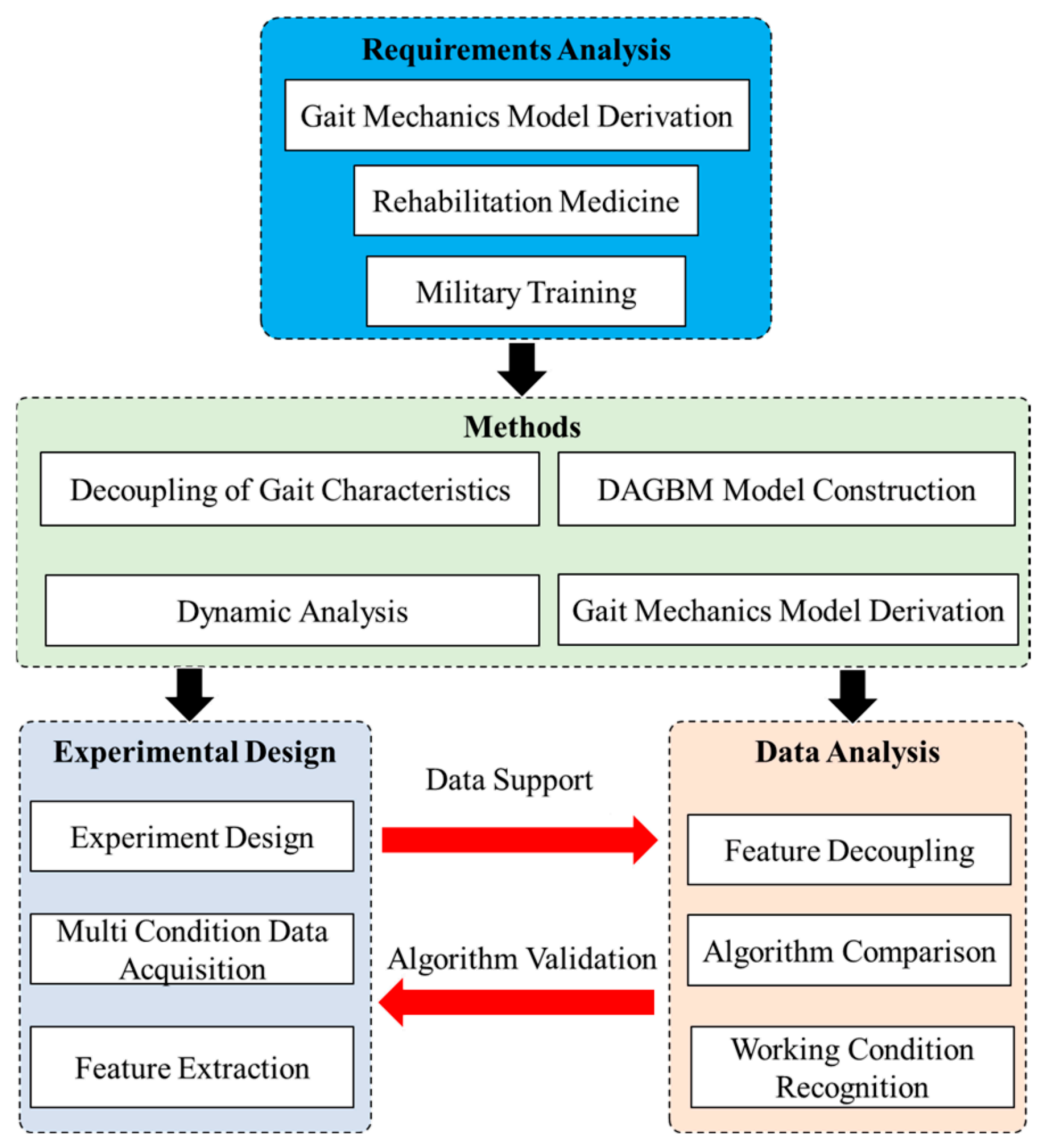
Overall research framework of this paper.

To solve the problems mentioned in the introduction, this paper establishes an innovative integrated framework consisting of theoretical support, system implementation, and experimental validation, which is shown in Figure 1. The core theoretical system for dual working condition gait analysis is established through the derivation of the gait mechanics model, system dynamic analysis, and research on the feature decoupling mechanism. Based on the theoretical indicators, 3D modeling, hardware processing, and system integration of the test bench are completed, and a specialized gait test platform that can cover the full gradient coupled working conditions of 0–60% gravity unloading and 0–30% body weight bearing is independently developed. Relying on this platform, multi-condition gait data acquisition is completed, gait feature decoupling and high-precision working condition recognition under coupled working conditions are realized, which provides a novel technical approach for astronaut walking state monitoring and gravity adaptation strategy research in deep-space exploration.

### 2.2. Dynamic Analysis of Gait Under Partial Body Weight Support–Weight-Bearing Conditions

The human lower limb is simplified as a 7-rigid-body model, including the pelvis, bilateral thighs, shanks, and feet. The inertial parameters of the rigid bodies are adopted from the internationally accepted standard human biomechanical model [11]. The planar motion of an arbitrary rigid body segment satisfies the Newton–Euler dynamic equation:

(1) $$\sum F=m_{i}a_{\mathrm{ci}}$$

(2) $$\sum M=I_{i}\alpha_{i}+r_{\mathrm{ci}}\times (m_{i}a_{\mathrm{ci}})$$

where *m_i_* is the mass of the rigid body segment; *a_ci_* is the centroid acceleration; *I_i_* is the moment of inertia about the center of mass; *α_i_* is the angular acceleration; *r_ci_* is the position vector from the joint center to the center of mass.

Substituting Equation (2) for the equivalent gravitational acceleration *g_eq_* and the load mass *m_load_* into the inverse dynamics framework yields the net joint moments.

*M_joint_* = *I_i_α_i_* + *m_i_*(*a_ci_* − *g_eq_*) × *r_ci_* + *M_grf_* + *M_load_* (3)

where *M_grf_* is the moment generated by the ground reaction force, and *M_load_* denotes the additional moment induced by the carried load.

In summary, the above model can quantitatively describe the effects of gravity and load on lower extremity dynamic characteristics.

### 2.3. Mechanical Analysis of the 3-Point Suspension Low-Gravity Simulation System

**Figure 2:**
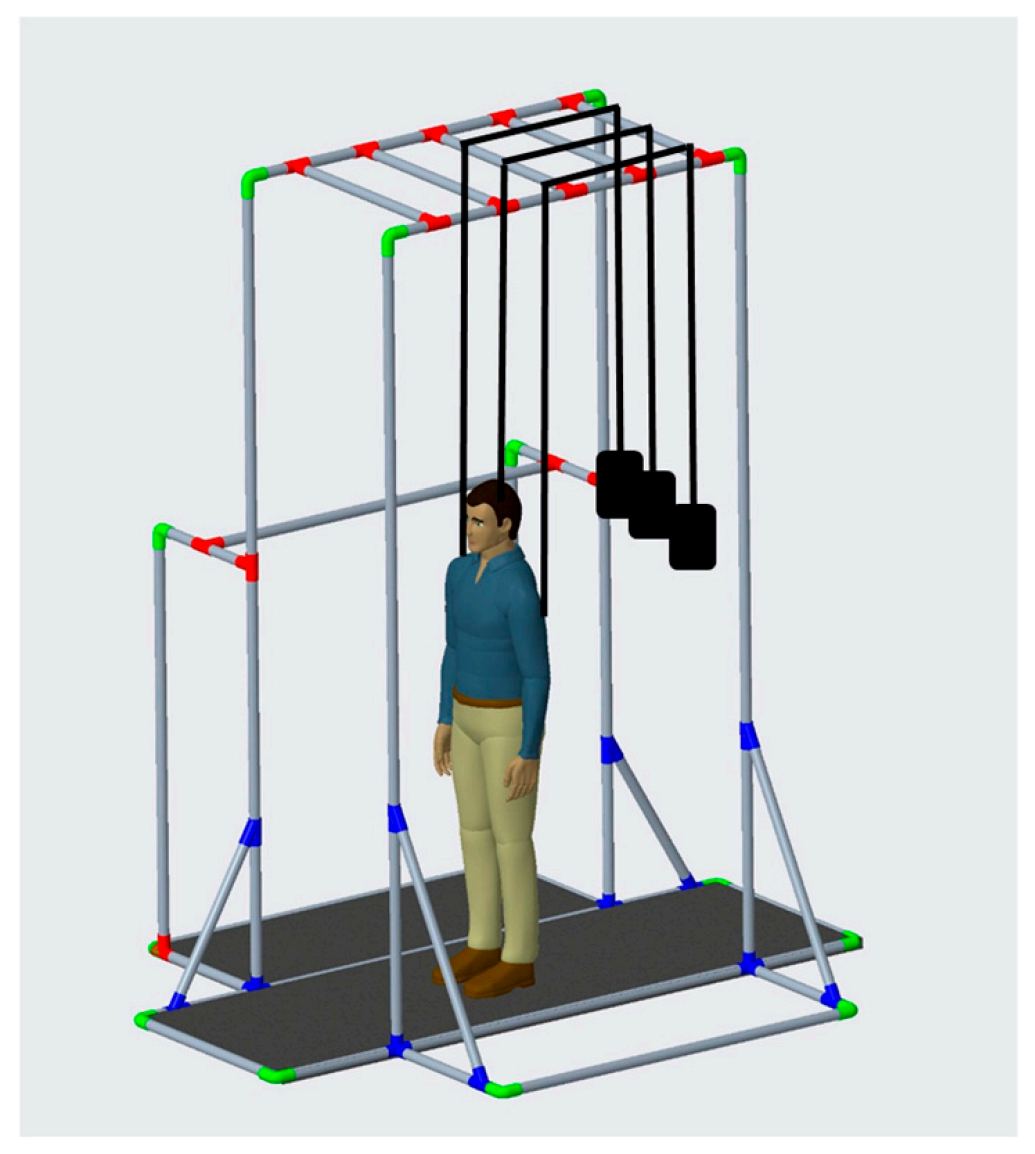
A 3D design of a 3-point suspension low-gravity test bench.

In the mechanical analysis of low-gravity simulation, the resultant suspension tension is determined solely by the total vertical upward force and is not directly related to the number of suspension points. Regardless of the suspension configuration adopted, the core objective of the system is to provide an equivalent total vertical upward tension to offset partial gravity [12]. Therefore, a 3-point suspension low-gravity experimental platform was constructed, as shown in Figure 2. Suspension points are located at the posterior back and bilateral waist. The yellow, green, and blue structures in the Figure 2 are steel pipe connectors for building the test platform. 

The system applies vertical upward tension to offset part of the subject’s body weight to achieve gravity unloading, thereby simulating a low-gravity state. Furthermore, the experimental platform does not restrict the horizontal degrees of freedom of walking, ensuring natural gait movement.

The force analysis of the subject in the vertical direction considers the following: the total vertical downward gravity *m_total_g* acting on the human body and the carried load, where the total mass *m_total_* is the sum of the human body mass *m_body_* and the load mass *m_load_*; the upward tension *F_s_* provided by the 3-point suspension system; and the vertical upward ground reaction force *F_N_* exerted by the treadmill on the human body. When the subject stands still or walks at a constant speed on the experimental platform, the forces in the vertical direction are in equilibrium, satisfying the static equilibrium relationship.

*F_s_* + *F_N_* = *m_total_g* (4)

where *g* is the standard gravitational acceleration.

To characterize the low-gravity simulation effect, the equivalent gravitational acceleration *g_eq_* is introduced, and the ground reaction force is equivalently expressed as:

*F_N_* = *m_total_g_eq_* (5)

Substituting into the above equation:

*F_s_* + *m_total_g_eq_* = *m_total_g* (6)

This equation is the core mechanical equilibrium equation of the low-gravity simulation system. In addition, let *η* denote the gravity unloading rate; we have:

*g_eq_* = *g*(1 − *η*) (7)

Then Equation (6) can be rewritten as:

*F_s_* = *η*(*m_body_* + *m_load_*)*g* (8)

Prior to the experiment, the actual tension was measured using a force sensor and compared with the theoretical value to ensure the system error was less than 2%. It is worth noting that Equations (4)–(8) describe quasi-static vertical force equilibrium under standing or cycle-averaged conditions of the suspension system. These formulas provide theoretical baseline values for average supported load and cannot directly predict instantaneous dynamic peak plantar pressure during human walking. Dynamic plantar pressure peaks are jointly modulated by human gait adjustment, muscle co-contraction, and movement adaptation under coupled unloading-load conditions, which cannot be fully explained by static force-balance derivation.

### 2.4. Variance Component Estimation and Feature Screening for Gait Features

In the coupled partial body weight support and weight-bearing environment, gait features are jointly affected by individual differences in physiological structure and external environmental variations [13]. To accurately extract the environment-sensitive features related to gravity and load, and eliminate the interference of individual differences in working condition recognition, a gait feature variance-component estimation followed by environment-sensitive feature screening is constructed in this paper to realize the effective separation of inherent individual features and environmental interference features. Firstly, the gait features of any subject are decomposed into 3 parts: the inherent individual component, the environment-sensitive component, and random noise. The model expression is as follows:

*x_i_*_,*f*_ = *β_i_*_,*f*_ + *γ_η_*_,*ω*,*f*_ + *ε_i_*_,*f*_ (9)

where *x_i,f_* denotes the *f*-dimensional gait feature of the *i*-th subject. *β_i,f_* is the inherent individual feature component, which is determined by the subject’s physiological attributes such as height, body weight, and limb proportions, and is not affected by external environmental factors such as gravity and weight bearing; *ε_i,f_* represents the *f*-dimensional measurement noise and random fluctuation component of the *i*-th subject, which mainly originates from the error of experimental equipment and minor fluctuations during the subject’s gait movement. *γ_η,ω,f_* is the environment-sensitive component, which is jointly affected by the gravity offset ratio *η* and the relative load ratio *ω*. This index is the core effective information for subsequent classification tasks, and the expression of the load ratio *ω* is as follows:

(10) $$\omega =\frac{m_{\mathrm{load}}}{m_{\mathrm{body}}}$$

In this paper, a linear mixed-effects model is adopted to estimate the variance of each feature component [14]. By defining the contribution degree of individual features and the contribution degree of environmental sensitivity, the influence degrees of individual differences and environmental changes on gait features are quantified [15,16,17]. Environmental sensitivity contribution degree: it characterizes the proportion of the influence of the environment-sensitive component on gait features, and its expression is as follows:

(11) $$R^{2}=\frac{\mathrm{Var}(\gamma_{\eta ,\omega ,f})}{\mathrm{Var}(\beta_{i,f})+\mathrm{Var}(\gamma_{\eta ,\omega ,f})+\mathrm{Var}(\varepsilon_{i,f})}$$

where *Var* denotes the variance of the corresponding component.

To improve the working condition classification accuracy and cross-individual generalization ability of the subsequent pattern recognition model, the initial gait feature set is screened according to the environmental sensitivity contribution degree *R*^2^. Based on the linear mixed-effects model, the environmental sensitivity contribution degree of each feature is quantified. Statistics show that the cumulative contribution degree of the top 80% of features reaches 92.7%, which can fully retain the working condition-sensitive information. These features resp ond significantly to changes in the gravity offset ratio *η* and weight-bearing ratio *ω*. The remaining 20% of features, for which the individual variance accounts for more than 75%, are eliminated. In this way, the interference of individual differences on working condition recognition is weakened, and the influence law of environmental changes on gait features is highlighted.

### 2.5. Construction of Domain-Adaptive Gradient Boosting Machine Model

Aiming at the problems of feature distribution shift caused by individual biomechanical differences and insufficient generalization ability under small-sample conditions in the recognition of weight reduction–load-carrying coupling conditions, this paper constructs a domain-adaptive gradient boosting machine (DAGBM) to realize high-precision classification of gravity offset levels, load-carrying levels, and coupled working conditions. Based on the gradient boosting framework, the model introduces a domain-adaptive regularization term and is complemented by an individual–environment feature decoupling method, which suppresses the classification interference induced by individual differences while retaining condition-sensitive features. The model features a simple structure and strong robustness. The gradient boosting machine constructs an additive ensemble model by iteratively training multiple CART regression trees, and its basic prediction formulation is given as follows:

(12) $$F(x)=F_{0}(x)+\eta \sum_{m=1}^{M}h_{m}(x)$$

where *F*_0_(*x*) is the initial value of the model; *η* denotes the learning rate; *M* represents the number of decision trees; *h_m_*(*x*) is the predictive output of an individual tree.

To mitigate the feature domain shift across subjects, this paper introduces the maximum mean discrepancy (MMD) domain-adaptive regularization term based on the traditional classification loss to construct the total loss function:

*L_total_* = *L_cls_* + *λ* × *L_MMD_* (13)

where *L_cls_* denotes the multi-class cross-entropy loss, which is employed to optimize the classification accuracy of working conditions; *L_MMD_* is used to reduce the feature distribution distance between subjects in the training set and test set; and *λ* represents the regularization balance coefficient that controls the constraint intensity of domain adaptation. It should be emphasized that the MMD regularization term in Equation (13) is not calculated based on fixed hand-crafted input features. Instead, MMD is computed over latent representations dynamically generated in each boosting iteration. After each new CART tree is trained, latent outputs of training samples are updated. The MMD loss penalizes inter-subject distribution discrepancy on these updated latent features, which further affects sample weights and guides tree-splitting criteria for the subsequent boosting round. After feature decoupling, the MMD of the cross-subject feature distribution decreases from 0.412 to 0.138, and the interference from individual differences is reduced by 66.5%, verifying the effectiveness of the decoupling operation.

Strict leave-one-subject-out (LOSO) cross-validation is adopted for model evaluation. Within each LOSO fold, all samples from one participant are held out as the exclusive test dataset, and samples from the remaining 19 participants constitute the training dataset. Critically, feature decoupling and screening, feature normalization, and Bayesian hyper-parameter optimization are executed independently using only training-set data in every fold; no data or statistics from the held-out test participant enter any part of the training pipeline. The MMD domain-alignment loss is calculated exclusively on training-subject samples. Data from the held-out test participant are never involved in MMD computation or model optimization. Therefore, the present evaluation corresponds to inductive cross-subject generalization rather than transductive domain adaptation. Hyperparameters such as learning rate, tree depth, and regularization coefficient are adaptively adjusted via Bayesian optimization. The model finally outputs the classification results of gravity offset levels, load-carrying levels, and 28 types of coupled working conditions. To support reproducibility, Table 1 lists the hyper-parameter search ranges of the proposed DAGBM model. The detailed pseudocode for the MMD-based domain-adaptive gradient-boosting update is provided in the Supplementary Material.

In this paper, accuracy, precision, recall, and *F*_1_ score are adopted to evaluate the performance of pattern recognition [18,19,20,21]. Accuracy *A_cc_* is the most intuitive and fundamental evaluation metric in multi-class classification tasks. It quantifies the overall recognition capability of the model across all classes, representing the ratio of correctly classified samples to the total number of samples [22]; precision *P_re_* represents the proportion of samples truly belonging to a certain class among those predicted as that class by the model [23]. It is used to evaluate the reliability of classification results and avoid misclassifying irrelevant samples as the target class; recall *R_ec_* refers to the proportion of samples that truly belong to a certain class and are correctly identified by the model [24]. It is employed to evaluate the model’s ability to fully detect target classes; *F*_1_ score is the harmonic mean of precision and recall, which comprehensively reflects the balanced performance of the model in terms of accuracy and completeness, avoiding evaluation bias caused by the preference of a single metric.

## 3. Experimental Design and Data Processing

### 3.1. Experimental Subjects

A total of 20 healthy adult subjects—10 males and 10 females—were recruited in this paper, with a gender ratio of 1:1 to balance the potential influence of gender differences on gait characteristics. The subjects were aged 22–33 years, with a mean age of 26.4 ± 3.2 years; the height ranged from 158 cm to 185 cm, with a mean value of 170.2 ± 8.7 cm; the body weight was 52–81 kg, with a mean value of 64.7 ± 9.3 kg; the BMI was 19.2–23.8 kg/m^2^, with a mean value of 21.5 ± 1.3 kg/m^2^, all within the normal BMI range for healthy Chinese adults.

All experiments designed in this paper are harmless to the human body. All subjects participated voluntarily and signed the informed consent form before the experiment, with a clear understanding of the experimental procedures, contents, potential risks during the experiment, and the right to withdraw at any time. This research was also approved by the Institutional Review Board of Shandong University of Aeronautics (Protocol code: 26092806, approval date: 28 September 2026).

### 3.2. Experimental Design

**Figure 3:**
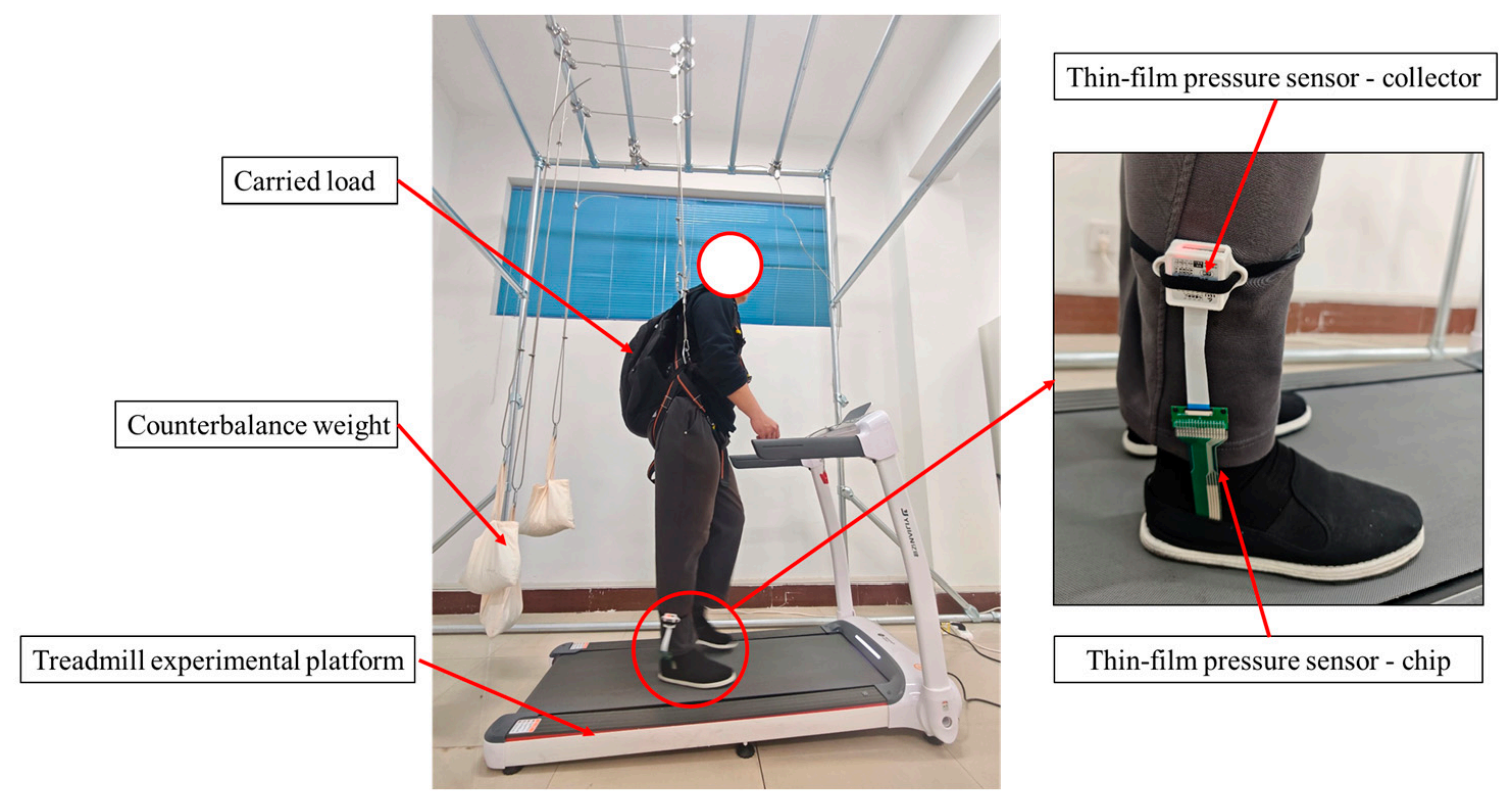
Body weight support–weight-bearing test bench.

The experimental system consists of a body-weight-support/weight-bearing test bench, core detection equipment, working condition simulation equipment, and data synchronization equipment. The site layout is shown in Figure 3. The three suspension points are arranged on the back and both sides of the waist, respectively. Steel cables are connected to counterbalance weights through fixed pulleys. To simulate practical application scenarios, the load mass is controlled by a backpack in this experiment. The counterbalance weights are small steel balls with a diameter of 1 cm, which can be adjusted precisely with an error ≤ 0.1 kg. This setup ensures the left–right balance of the subject and theoretically supports a gravity offset range of 0% to 100%.

A flexible pressure sensor array (Model: ES48P) is selected as the pressure acquisition device, featuring an array density of 4 × 12, a detection range of 0–200 kPa, a sampling frequency of 100 Hz, and a thickness of 0.2 mm. The sensor system is composed of chips, a collector, a Bluetooth receiver, and a host computer. The chips can be closely attached to the forefoot, midfoot, and hindfoot regions of the plantar surface. Before the experiment, a standard pressure calibrator was used for static and dynamic calibration, with a calibration error ≤ 2.5%. In this paper, a 48-point array plantar pressure sensor attached to the right foot was used to collect time-series plantar pressure data during walking.

All subjects wore uniform inelastic thin-soled flat experimental shoes before the test to eliminate the interference of shoe type and sole hardness on plantar pressure measurement, and the sensor array was attached to the corresponding plantar position inside the shoes. The sensor was fixed with hypoallergenic medical silicone breathable adhesive to ensure no displacement, wrinkling, or suspension during the test. Before the test, the tightness of the suspension straps and the load position were adjusted one by one to ensure that the subjects had no sense of restraint or discomfort, and to avoid center of gravity offset and abnormal gait caused by unilateral load bearing. Apart from static calibration, additional tests were performed to evaluate sensor repeatability, time drift, and dynamic measurement performance under gait-like loading conditions, which support the reliability of dynamic plantar-pressure measurement in this study.

### 3.3. Data Acquisition

To eliminate the interference of walking speed differences on experimental results, a constant walking speed of 3 km/h was adopted for all working condition tests in this paper. Before the formal test, the subjects walked continuously at this fixed speed for 5 min under the standard flat ground condition with 0° slope, 0% body weight support, and 0% weight bearing to complete the adaptation training for the experimental environment and equipment. It was confirmed that there were no symptoms of discomfort such as dizziness or lower limb fatigue, and the stability of the data acquisition system was verified simultaneously.

Prior to formal data acquisition, multi-dimensional calibration of the entire test system was first performed to ensure measurement accuracy and data synchronization. The subjects kept their plantar surfaces unloaded and suspended to collect baseline zero data. Full range calibration was completed using standard steel balls to ensure that the linear correlation coefficient R^2^ between the sensor measurements and the standard load was greater than 0.99, which met the accuracy requirements for dynamic gait pressure measurement. Subsequently, the suspension tension of the gravity offset system, the loading mass of the load-bearing vest, and the treadmill slope parameters were calibrated one by one. The control error of all parameters was controlled within ±1% to ensure that the working condition settings were completely consistent with the experimental design. Finally, an external synchronous trigger device was used to achieve precise timestamp alignment of the plantar pressure acquisition system.

The independent variables of the experiment were body weight support and weight bearing, while the dependent variable (acquired data) was the plantar pressure parameter. The specific level settings and their corresponding basis are shown in Table 2.

**Figure 4:**
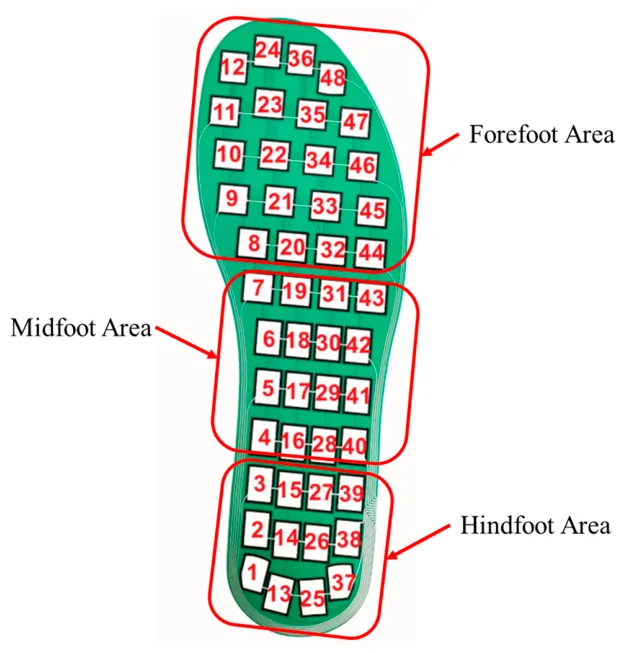
Distribution of measuring points of the plantar pressure sensor.

To eliminate the interference of measurement noise and random fluctuations from a single sensing unit, and conform to the load-bearing characteristics of the plantar anatomical structure, all sensing units were divided into three plantar functional regions according to the row numbers of the sensor array, as shown in Figure 4. Numbers 1–48 in the Figure 4 denote the points of the sensor, which can simultaneously collect data from 48 positions.

The hindfoot region is the heel load-bearing region. It corresponds to 12 sensing units in rows 1–3 of the array, covering the core load-bearing area of the calcaneus and bearing the impact load during the gait heel strike phase.

The midfoot region is the arch support region. It corresponds to 16 sensing units in rows 4–7 of the array, covering the arch support structure and performing the functions of load transmission and buffering during the mid-stance phase of gait.

The forefoot region is the area involved in the forefoot push-off. It corresponds to 20 sensing units in rows 8–12 of the array, covers the load-bearing areas of the metatarsals and toes, and participates in force generation and posture control during the gait push-off phase.

Under each working condition, data acquisition was initiated after the subjects walked stably at the preset speed for 1 min, with an acquisition duration of 3 min to ensure that no less than 100 valid gait cycles were obtained. Tests were conducted on 20 subjects under 28 working conditions, yielding a total of 20 × 28 × 100 = 56,000 valid samples. During the acquisition process, the treadmill safety handrails were enabled throughout the test, and two experimenters were assigned to monitor to prevent accidents. For rest intervals between working conditions, the subjects rested for 2 min after each test and 5 min after every four tests to ensure complete recovery from muscle fatigue. Gait cycles were segmented based on foot-contact timestamps detected from plantar-pressure time-series signals. The 3-σ criterion was adopted for outlier rejection. Missing data frames were directly discarded without data imputation, as missing samples were extremely rare in the present dataset.

During data screening, unstable gait data in the starting and stopping phases were eliminated, and only the stable data in the middle 3 min were retained. Meanwhile, the method was adopted to process outliers in the data. Four core gait parameters were extracted, including peak pressure, pressure–time integral, regional pressure ratio, and standard deviation of the center of pressure (CoP) trajectory. All model-related preprocessing operations, including normalization and feature selection, are performed fold-wise under the LOSO framework to avoid data leakage across participants.

### 3.4. Feature Extraction

#### 3.4.1. Regional Instantaneous Peak Pressure

Regional instantaneous peak pressure refers to the maximum value of the instantaneous pressure values collected by all pressure sensing units in the target plantar region at each sampling moment of the gait cycle, and this maximum value is defined as the regional instantaneous peak pressure at that moment [25]. The regional instantaneous peak pressures at each moment are arranged in chronological order to form a continuous time-series curve of peak pressure, which is used to characterize the dynamic load variation of the region throughout the gait cycle. The calculation formula is as follows:

(14) $$P_{\mathrm{inst}-\mathrm{peak}}(t_{j})=\max_{i\in S}p_{i}(t_{j})$$

where *t_j_* denotes the *j*-th sampling moment; *S* denotes the set of all sensors in the target plantar region; *p_i_*(*t_j_*) denotes the instantaneous pressure of the *i*-th sensor in the region at moment *t_j_*.

#### 3.4.2. Regional Pressure–Time Integral

The pressure–time integral (PTI) reflects the cumulative comprehensive load of pressure and action time in a local region (kPa·s) [26]. It reflects excellent individual differences, has gait pattern discrimination capability, and can be used as a stable and effective feature for gait pattern recognition and classification tasks. The calculation formula is as follows:

(15) $$\mathrm{PT}I_{i}\;=\;\Delta t\times \sum_{k=1}^{M}P_{i,k}$$

where △*t* is the sampling time interval; *M* denotes the number of sampling points in a single gait cycle. Then

(16) $$M=\frac{\left(t_{e}-t_{s}\right)}{\Delta t}$$

To calculate the regional PTI, the values of all sensors within the region shall be summed up.

#### 3.4.3. Regional Pressure Proportion

This parameter eliminates instantaneous fluctuations during gait and reduces individual experimental differences; thus, it can better characterize the long-term load-bearing features of plantar regions. The calculation formula is as follows:

(17) $${\bar{R}}_{\mathrm{area}}=\frac{\sum_{i\in \mathrm{area}}\mathrm{PT}I_{i}}{\sum_{j=1}^{N_{\mathrm{total}}}\mathrm{PT}I_{j}}\times 100\%$$

where *area* denotes the defined target region; *N_total_* is the total number of sensors (*N* = 48).

#### 3.4.4. Standard Deviation of the Whole Foot Center of Pressure (CoP) Trajectory

This measures the degree to which the *x* and *y* coordinates of the CoP deviate from the mean value within a single gait cycle or the entire acquisition period (reflecting the stability of the CoP trajectory; a smaller value indicates higher stability) [27,28,29]. First, the CoP coordinates at each time point are calculated as follows:

(18) $$x_{\mathrm{CoP}}(t)=\frac{\sum_{i=1}^{N_{\mathrm{total}}}x_{i}\cdot P_{i,t}}{\sum_{i=1}^{N_{\mathrm{total}}}P_{i,t}}$$

(19) $$y_{\mathrm{CoP}}(t)=\frac{\sum_{i=1}^{N_{\mathrm{total}}}y_{i}\cdot P_{i,t}}{\sum_{i=1}^{N_{\mathrm{total}}}P_{i,t}}$$

where *x_i_* and *y_i_* are the physical coordinates of the *i*-th sensor.

Then the standard deviation of the CoP trajectory(cm) is calculated.

(20) $$SD_{x,\mathrm{CoP}}=\sqrt{\frac{1}{K-1}\sum_{K=1}^{K}{\left(x_{\mathrm{CoP}}(k)-{\bar{x}}_{\mathrm{CoP}}\right)}^{2}}$$

(21) $$SD_{y,\mathrm{CoP}}=\sqrt{\frac{1}{K-1}\sum_{K=1}^{K}{\left(y_{\mathrm{CoP}}(k)-{\bar{y}}_{\mathrm{CoP}}\right)}^{2}}$$

where *K* is the number of sampling points within the calculation range, such as sampling points in a single gait cycle or all valid gait sampling points within 3 min. To reflect the overall fluctuation, the combined standard deviation can be calculated. The formula is as follows:

(22) $$SD_{\mathrm{CoP}}=\sqrt{SD_{x,\mathrm{CoP}}^{2}+SD_{y,\mathrm{CoP}}^{2}}$$

According to the physical layout of the sensor, the measuring point labeled No. 1 of the plantar pressure sensor in Figure 4 is set as the coordinate origin with coordinates (0, 0). Based on the physical positions of the sensors, the coordinates of all 48 sampling points are calculated, and the results are presented as follows in Table 3:

## 4. Analysis of Variation Regularity of Gait Characteristic Parameters

Prior to the experiment, calibration results showed that within the pressure range of 0–200 kPa, the flexible thin-film pressure sensor exhibited a linear correlation coefficient R^2^ = 0.996 between measured values and standard pressure values, with an average error of 2.3%, meeting the accuracy requirements for plantar pressure measurement. In dynamic tests, the sensor could accurately capture key gait events such as heel strike and toe-off, verifying the validity of the acquired data.

### 4.1. Effect of Body Weight Support on the Instantaneous Peak Plantar Pressure

**Figure 5:**
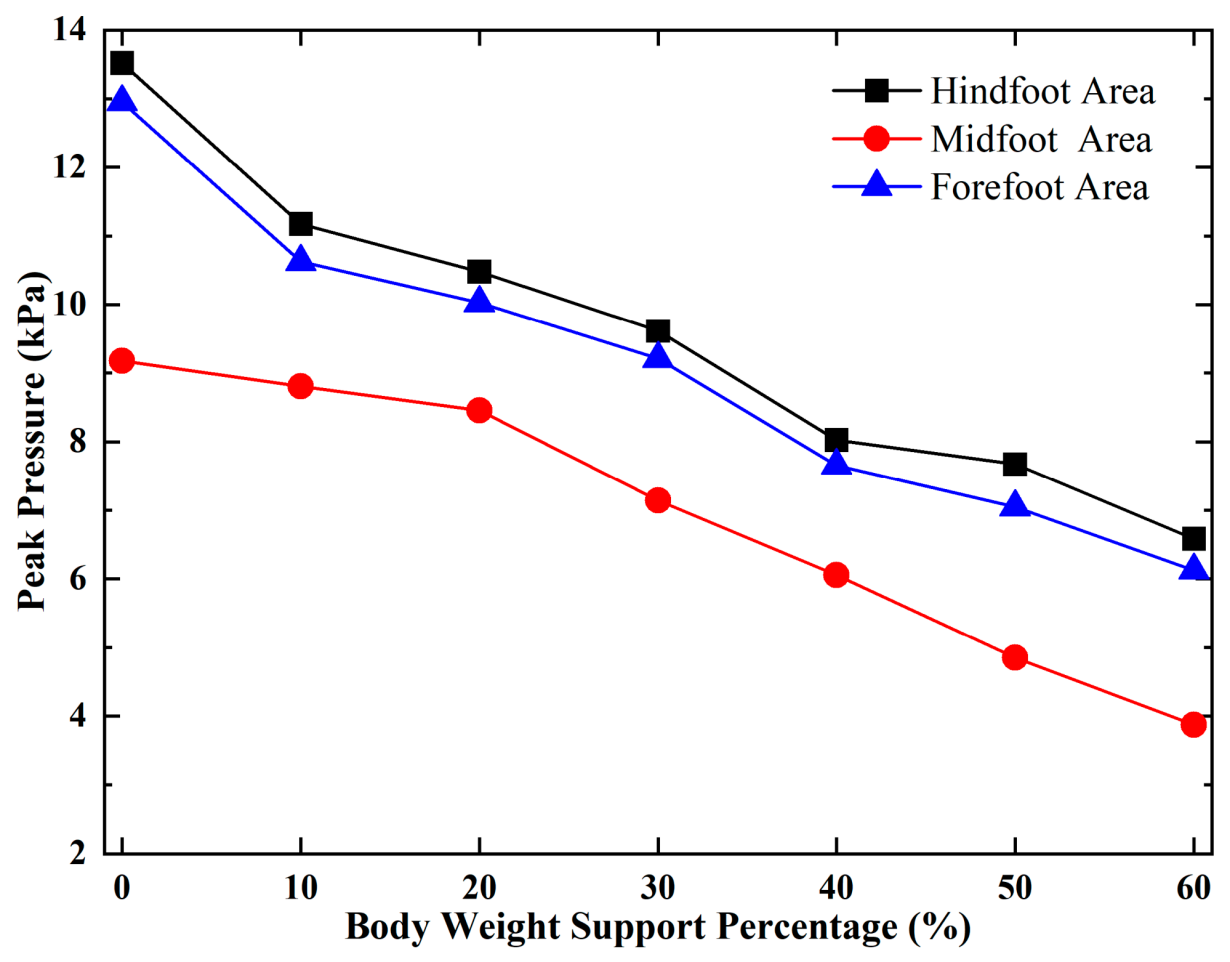
Effect of body weight support on the instantaneous peak plantar pressure.

When humans walk under weight-reduction conditions, the variation in peak pressure across different plantar regions is shown in Figure 5. This figure depicts the influence of weight reduction percentage (0–60%) on the peak plantar pressure (kPa) in three anatomical regions: hindfoot, midfoot, and forefoot area. The peak pressure in all three regions shows a continuous monotonic decreasing trend with increasing weight-bearing ratio, while a stable pressure hierarchy is maintained throughout. Under the baseline condition (0% weight bearing), the hindfoot area exhibits the highest peak pressure, followed by the forefoot area, and the midfoot area shows the lowest pressure. At a body weight support percentage of 60%, the pressure in all three regions drops to the minimum value during the test. Among them, the midfoot area pressure has the largest descending slope and the most significant attenuation amplitude; the pressure attenuation rates of the hindfoot and forefoot areas are relatively gentle. The three curves show no crossing or inflection points, indicating that weight reduction only regulates the magnitude of plantar pressure without altering the spatial distribution characteristics of plantar loading.

The peak plantar pressure essentially originates from the vertical ground reaction force induced by body weight. The reduction in body weight directly decreases the axial load during the gait cycle, which constitutes the fundamental mechanical cause for the synchronous pressure decline in all three regions. This pattern is unaffected by the anatomical differences of plantar regions and represents a characteristic of human weight-bearing biomechanics. In normal gait, the hindfoot serves as the initial contact region and bears the maximum load during the impact phase; the forefoot participates in the push-off and propulsion phase, undertaking the dynamic load of propulsion; the midfoot is dominated by the elastic structure of the foot arch, whose core function is cushioning and shock absorption, thus naturally bearing a low load. The midfoot is mainly composed of soft tissues, ligaments, and the elastic foot arch. Its viscoelastic tissues are far more sensitive to load variations than rigid structures, leading to more pronounced stress release and thus a faster pressure drop with decreasing body weight. In contrast, the hindfoot and forefoot are primarily loaded by cortical bone and dense ligaments, featuring high structural rigidity and mechanical stability, resulting in a gentler attenuation amplitude of pressure with changes in body weight.

### 4.2. Effect of Weight Bearing on the Instantaneous Peak Plantar Pressure

**Figure 6:**
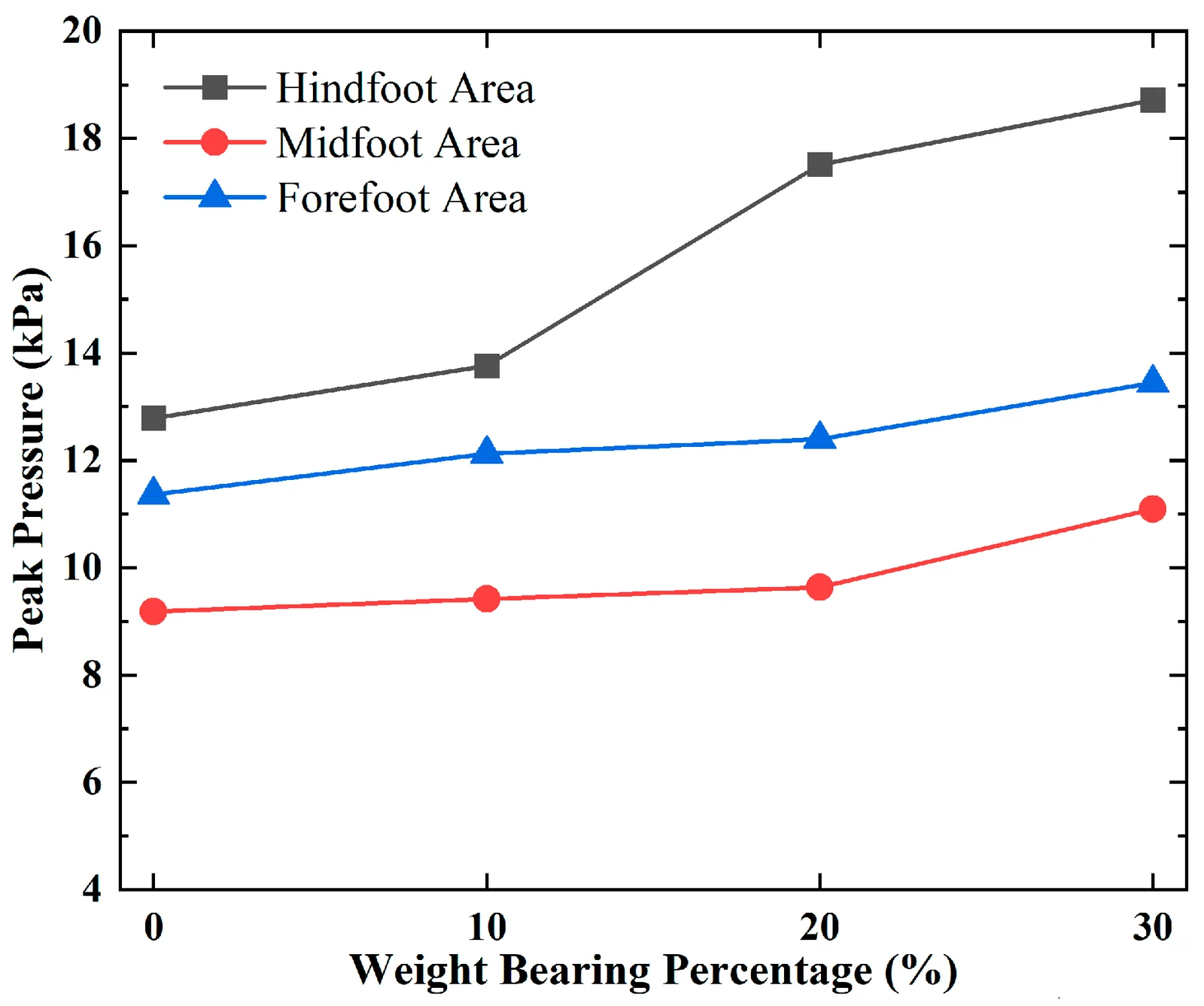
Effect of weight bearing on the instantaneous peak plantar pressure.

Figure 6 illustrates the variation in peak plantar pressure (kPa) across the hindfoot, midfoot, and forefoot under weight-bearing ratios increasing from 0% to 30%. Peak pressure in all three regions shows a continuous upward trend with increasing load proportion. The hindfoot exhibits the most pronounced pressure increase and maintains the highest value throughout the test. The forefoot pressure rises steadily, with its magnitude consistently lower than that of the hindfoot but higher than that of the midfoot. The midfoot pressure increases gently under low-load conditions (0–20%) and accelerates from 20% to 30%, while remaining at the lowest pressure level overall.

The increase in load percentage directly elevates the axial weight-bearing load, which constitutes the fundamental cause of the synchronous rise in pressure across all three regions. This pattern is independent of anatomical differences in the plantar areas and represents a characteristic of human weight-bearing biomechanics. In normal gait, the hindfoot serves as the initial contact and primary weight-bearing structure, undertaking the majority of static and dynamic loads. Composed mainly of rigid osseous structures, the hindfoot exhibits no obvious buffering or attenuation in stress transmission, thus showing the highest sensitivity to load variations and the most pronounced pressure increase, remaining the dominant load-bearing region of the foot. The midfoot constitutes the core area of the elastic foot arch, whose primary function is cushioning and shock absorption. In the low-load stage (0–20%), it can dissipate stress through soft tissue deformation, resulting in a gentle pressure increase. Once the load exceeds 20%, the buffering capacity of the foot arch becomes saturated, leading to direct stress transmission and accelerated pressure growth, which conforms to the mechanical properties of viscoelastic tissues.

### 4.3. Analysis of Instantaneous Peak Plantar Pressure Variation Under Two-Factor Coupling Conditions

**Figure 7:**
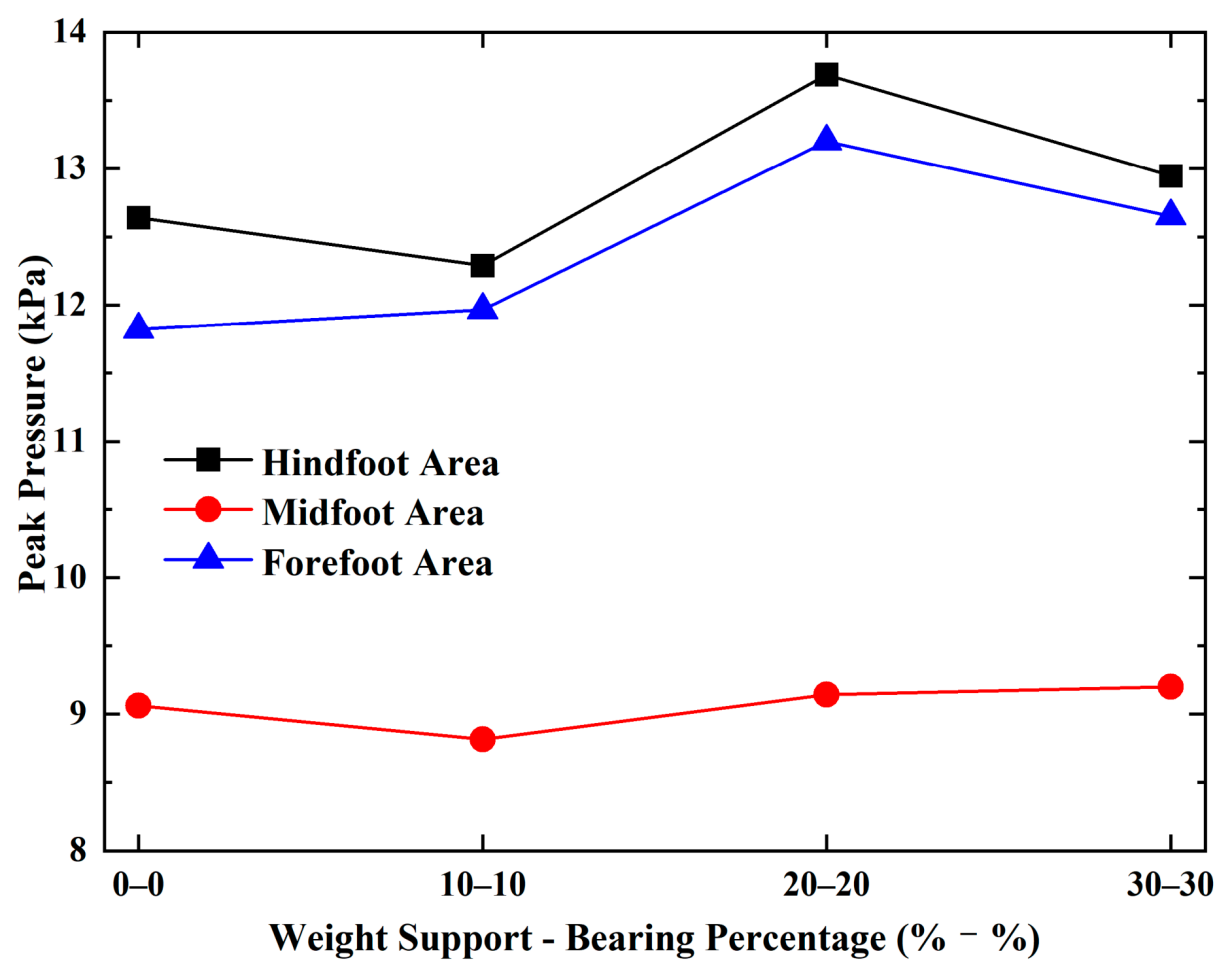
Variation of regional plantar-pressure peaks under representative diagonal coupled conditions of 2 factors.

Figure 7 illustrates the variation pattern of peak plantar pressure in the hindfoot, midfoot, and forefoot under equal proportion coupled conditions of body weight support and weight bearing (ranging from 0–0 to 30–30). A linear mixed-effects model (LMM) was further applied to statistically infer the effects of body weight support, load carriage, and their two-way interaction on peak plantar pressure. Participant identity was set as a random intercept to account for within-participant dependence introduced by repeated measurements. The LMM results indicate that during the gradient change of the coupled load, the variation trends of peak pressure in the three plantar regions were highly synchronized. Specifically, the pressure decreased slightly from the 0–0 to 10–10 condition, increased sharply to reach the maximum value across all working conditions from 10–10 to 20–20, and then showed a slow downward trend from 20–20 to 30–30. This figure also shows that under all test conditions, the hindfoot consistently exhibited the highest pressure, followed by the forefoot, while the midfoot maintained the lowest pressure with the gentlest variation. No crossing or abrupt abnormal changes were observed in the three curves, indicating that the coupled load only modulates the magnitude of plantar pressure without disrupting the inherent loading distribution pattern of the plantar region.

This occurs because the unloading effect of mild weight reduction dominates, resulting in a slight decrease in plantar load and a small drop in pressure. As the mechanical effect imposed by the external load exceeds the unloading effect of weight reduction, the net increment in vertical plantar load increases significantly, stress accumulates rapidly, and pressure reaches its peak. At a high body weight support ratio, the buffering and unloading effect is strengthened, offsetting part of the external load; consequently, the net plantar load decreases slowly, accompanied by a decline in pressure.

### 4.4. Variation of Pressure–Time Integral in Each Region Under Baseline Condition

**Figure 8:**
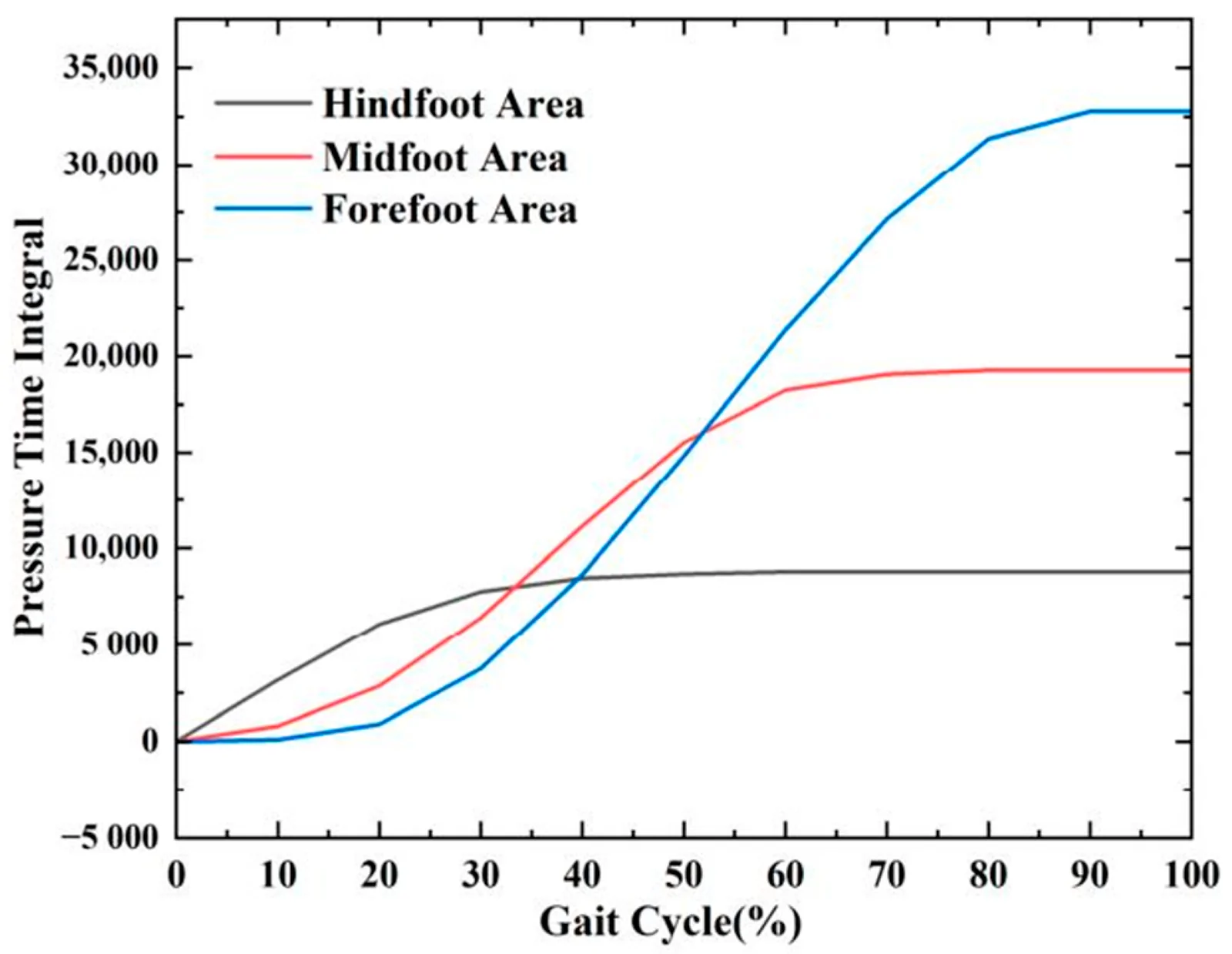
Variation of pressure–time integral in each region under baseline condition.

Figure 8 shows the dynamic variation of pressure–time integral in the hindfoot, midfoot, and forefoot during a complete gait cycle. In the early stage of the gait cycle (0–30%), the hindfoot first generates cumulative load with the fastest rising rate, the midfoot load increases slowly, and almost no load accumulation occurs in the forefoot. In the middle stage (30–60%), the load in all three regions rises rapidly, with crossing of the curves, and the dominant load-bearing region shifts from the hindfoot to the midfoot and forefoot. In the late stage (60–100%), the hindfoot load first enters a plateau and remains stable, the growth rate of midfoot load slows down and tends to saturate, and the forefoot load continues to rise to the maximum value of the whole cycle. Finally, a distribution pattern is formed where the forefoot has the highest cumulative load, followed by the midfoot, with the hindfoot having the lowest.

The dynamic variation of the pressure–time integral (PTI) in the hindfoot, midfoot, and forefoot, as shown in the figure, is essentially a direct reflection of plantar load transmission from posterior to anterior during the stance phase of human gait. In the early gait stage (0–30%), the hindfoot strikes the ground first and bears the initial impact load, leading to a rapid increase in cumulative load, while the midfoot undergoes only a gradual transition and the forefoot remains unloaded. In the middle stage (30–60%), with the forward shift of the body’s center of gravity, the midfoot and forefoot successively participate in weight bearing, and the dominant load-bearing region shifts from the hindfoot to the midfoot and forefoot, resulting in the crossing of the curves. In the late stage (60–100%), the hindfoot lifts off the ground, the midfoot stabilizes after completing load transmission, and the forefoot sustains a continuous rise in cumulative load to the maximum value of the entire cycle due to undertaking the additional dynamic load for push-off and propulsion. This finally forms a distribution characteristic of the highest cumulative load in the forefoot, followed by the midfoot, and the lowest in the hindfoot, which fully reflects the biomechanical transmission logic of hindfoot contact–midfoot transition–forefoot push-off.

### 4.5. Analysis of Pressure Proportion in Each Region Under Baseline Condition

**Figure 9:**
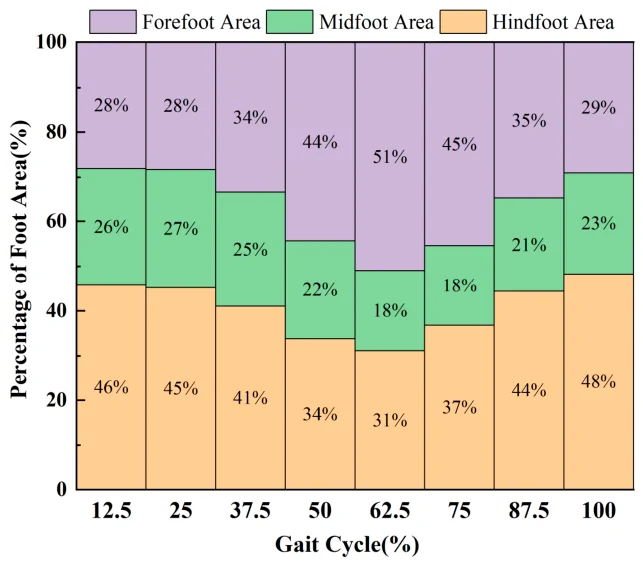
Pressure proportion in each region under baseline condition.

Figure 9 shows the distribution of the relative proportion of plantar pressure in the forefoot, midfoot, and hindfoot during 12.5–100% of the gait cycle under the baseline condition. In the early stage of the gait cycle (12.5%, 25%), the hindfoot accounts for the highest pressure proportion (46%, 45%) among the three regions, while the forefoot and midfoot show relatively balanced and low proportions. In the middle stage (37.5–62.5%), the proportion of the hindfoot decreases continuously; that of the forefoot increases significantly and reaches the peak value of 51% at 62.5%, making it the dominant pressure region, and the proportion of the midfoot exhibits a steady downward trend. In the late stage (75–100%), the proportion of the forefoot gradually declines and that of the hindfoot rebounds, with the hindfoot proportion recovering to 48% at 100%, and the proportions of the three regions return to a relatively balanced state. The sum of the pressure proportions of the three regions remains constant at 100% throughout the cycle, and the load distribution transitions smoothly without abnormal fluctuations.

In the initial stage of gait, the heel makes contact with the ground first, and the hindfoot serves as the only load-bearing structure in full contact with the ground, concentrating the vertical ground reaction force on the hindfoot. The forefoot and midfoot are not yet fully in contact with the ground, resulting in a small force-bearing area and low load. Therefore, the pressure proportion of the hindfoot is significantly higher than that of the other two regions, which conforms to the physical law of contact-based load bearing. The middle stage of gait enters the full foot support phase, where the plantar surface is fully in contact with the ground, and the load is physiologically transmitted from the hindfoot to the forefoot along the foot arch.

With the progression of gait and the forward shift of the body’s center of gravity, the forefoot gradually undertakes the core load of propulsion, with synchronous increases in force intensity and duration, leading to a rapid rise in its pressure proportion. The hindfoot gradually withdraws from the primary load-bearing position, with a continuous decline in its proportion. As the load transition zone, the midfoot shows a steady attenuation in its proportion. At the final stage of gait, after the forefoot completes push-off, the gait enters the heel contact reset phase; the body’s center of gravity falls back, and the hindfoot resumes bearing the main load, causing its proportion to rebound. The forefoot exits the high load state after completing propulsion, and its proportion naturally declines. The midfoot acts as an elastic cushioning structure throughout the cycle without bearing the core load, so its proportion remains stably low with no large fluctuations.

## 5. Algorithm Comparison and Pattern Recognition Result Analysis

### 5.1. Pattern Recognition Results

To verify the effectiveness of the proposed DAGBM in identifying body weight support–weight-bearing coupling conditions, three tasks are implemented in this section: body weight support level recognition, weight-bearing ratio level recognition, and comprehensive recognition of 28 types of coupling conditions. The comparative analysis is performed against four classical algorithms: SGBoost, random forest, support vector machine (SVM), and vanilla XGBoost. To avoid performance discrepancies caused by differences in data magnitude, the Min-Max normalization method is first used to normalize the dataset. Cross-subject independent tests are also conducted to validate the model’s generalization ability, with evaluation metrics consisting of accuracy, precision, recall, and F1 score as defined in Section 2.5. For fair comparison, all competing baseline models, including XGBoost, SGBoost, random forest, and SVM, adopt identical experimental configurations. They take the same feature set screened by mixed-effects feature-decoupling, apply the same Min-Max normalization, and are optimized via the same Bayesian hyper-parameter tuning framework.

### 5.2. Single Condition Level Recognition

Table 4 presents the single-condition classification accuracy (%) of different algorithms for the seven-class body weight support (BWS) task and the four-class weight-bearing level task. Comparative experimental outcomes indicate that our proposed DAGBM model yields the optimal recognition performance on both independent classification tasks. For the identification of different BWS levels, the DAGBM reaches an accuracy of 96.8%, while the recognition accuracy for weight-bearing levels is recorded at 97.4%. All contrast algorithms (SGBoost, RF, SVM, and XGBoost) produce inferior classification results on both tasks. Such obvious accuracy advantages verify that the domain-adaptive mechanism embedded in DAGBM can effectively extract discriminative gait features and substantially improve the recognition precision of single body weight support or weight-bearing working conditions compared with generic traditional machine learning models.

This is because the domain-adaptive regularization effectively weakens the feature shift caused by individual physiological differences, enabling the model to focus more on environment-sensitive features induced by gravity and weight bearing, thus forming a synergistic optimization effect with the feature decoupling method in Section 2.4.

### 5.3. Coupling Condition Level Recognition

For the 28 fine-grained and complex working conditions formed by the coupling of gravity offset and load carrying, the comprehensive recognition performance of each algorithm is presented in Table 5. The results demonstrate that the performance of traditional algorithms decreases significantly in the fine-grained classification task, with the accuracy of SGBoost being only 82.6%. In contrast, the proposed DAGBM model achieves a coupling condition recognition accuracy of 94.3% and an F1 score of 94.1%, representing an improvement of 2.8% over XGBoost and 11.7% over SGBoost. These results verify that the model possesses stronger feature discrimination ability and robustness in coupling scenarios with blurred class boundaries and weak feature differences.

**Figure 10:**
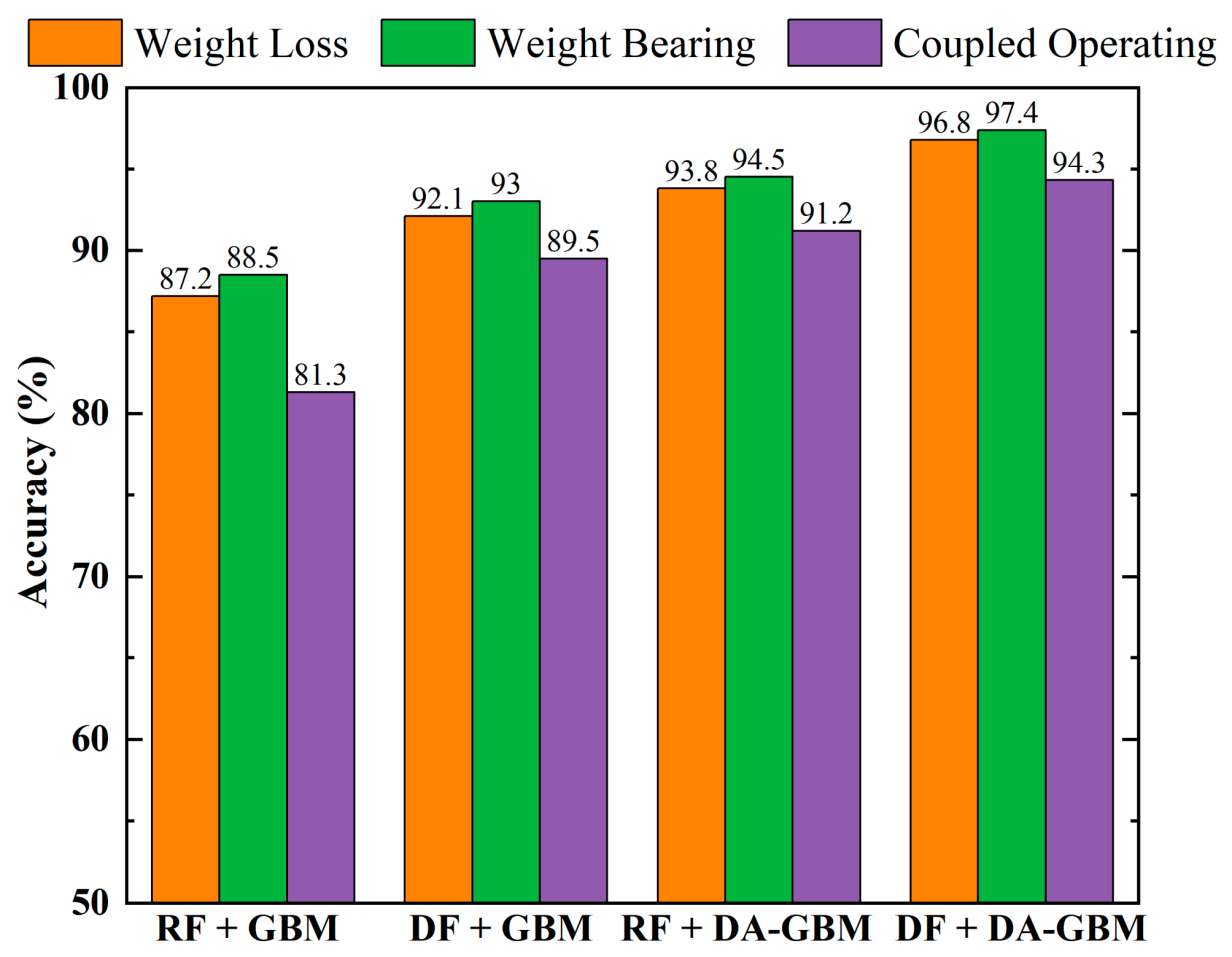
Comparison of ablation experiment results.

To verify the individual contributions and synergistic effects of the individual–environment feature decoupling module and the domain-adaptive gradient boosting module on the final recognition performance, four groups of controlled ablation experiments are designed in this paper: Raw features + traditional GBM, abbreviated as RF + GBM; decoupled features + traditional GBM, abbreviated as DF + GBM; raw features + DAGBM, abbreviated as RF + DAGBM; decoupled features + DAGBM, abbreviated as DF + DAGBM, which is the model proposed in this paper. By controlling a single variable, the improvements in recognition accuracy of body weight support levels, weight-bearing ratio levels, and 28-class coupled working conditions brought by feature decoupling and domain adaptation are quantified separately. The experimental results are shown in Figure 10.

It can be seen from the ablation experiment results that introducing individual–environment feature decoupling alone increases the recognition accuracy of coupled working conditions from 81.3% to 89.5%, with an absolute gain of 8.2%. This effectively eliminates feature redundancy and distribution shift caused by individual physiological differences, and significantly enhances the sensitivity and discriminability of gait features to weight-bearing and body weight support coupled conditions. Introducing the DAGBM domain adaptation mechanism alone improves the recognition accuracy of coupled working conditions from 81.3% to 91.2%, with an absolute gain of 9.9%. This effectively reduces cross-subject feature distribution differences and improves the model’s generalization ability under small-sample and fine-grained working conditions. The complete model combining feature decoupling and DAGBM proposed in this paper achieves the best recognition performance, with an improvement of 13.0% on the 28-class coupling task compared with the baseline GBM model.

This verifies that there is an optimization effect between the two modules. Feature decoupling can provide the model with highly robust environment-sensitive features, while domain adaptation can further suppress the interference of individual differences. The combination of the two enables high-precision and high-stability recognition.

### 5.4. Cross-Subject Generalization Performance Analysis

**Figure 11:**
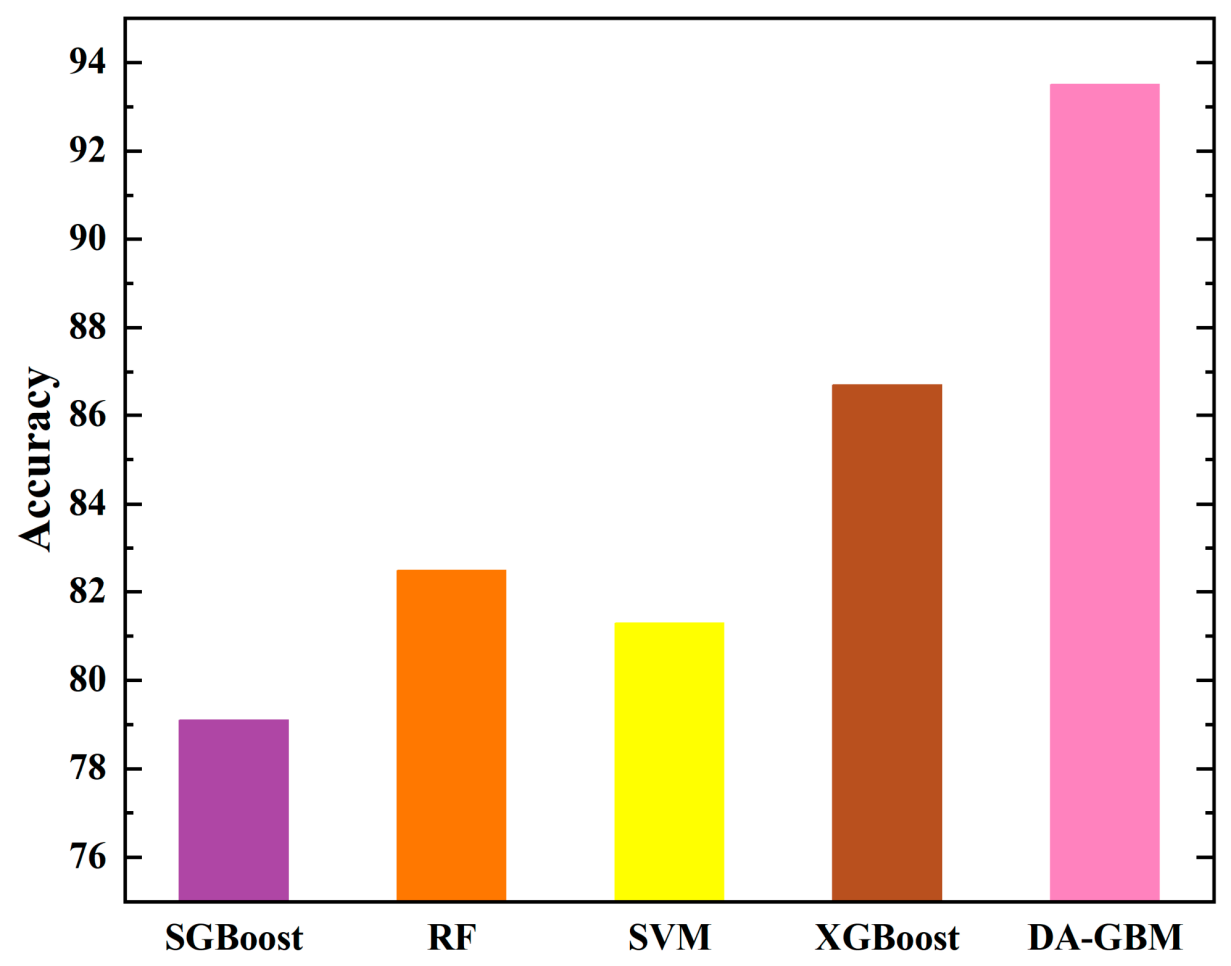
Comparison of cross-subject accuracy among different algorithms.

To further verify the engineering applicability of the model in real-world wearable scenarios, leave-one-subject-out cross-validation is adopted in this paper to conduct cross-individual generalization tests, with the results shown in Figure 11.

The cross-individual accuracies of traditional SGBoost and XGBoost drop to 79.1% and 86.7%, respectively, indicating that individual differences exert an impact on recognition accuracy. In contrast, the cross-subject recognition accuracy of the proposed DAGBM model remains at 93.5% with the minimum performance degradation, demonstrating that the domain-adaptive constraint can effectively eliminate feature distribution differences among different subjects and endow the model with stronger generalization ability and practical application value.

## 6. Conclusions and Outlook

To address the need for accurate gait recognition under body weight support–weight-bearing coupling conditions in scenarios such as deep-space exploration and rehabilitation training, this paper constructs a wearable plantar pressure acquisition system based on 48-channel flexible thin-film pressure sensors. The spatiotemporal distribution characteristics of human plantar pressure under multi-gradient coupling conditions are systematically investigated, and a gait recognition method combining individual–environment feature decoupling with a domain-adaptive gradient boosting machine (DAGBM) is proposed. The proposed method realizes high-precision classification of gravity offset levels, load-bearing ratio levels, and coupled working conditions.

The results demonstrate that the plantar region aggregated peak pressure, pressure–time integral (PTI), regional pressure proportion, and center of pressure (CoP) trajectory features can effectively quantify differences in gait mechanics under coupled conditions. Body weight support and weight bearing regulate the plantar pressure amplitude through unloading and loading effects, respectively, without changing the inherent plantar force distribution pattern. The constructed feature decoupling model can effectively separate individual physiological differences from environmental working condition features, significantly reduce the feature distribution shift across subjects, and improve the working condition sensitivity and robustness of features.

In terms of pattern recognition performance, the DAGBM model achieves recognition accuracies of 96.8% and 97.4% for seven-class gravity offset levels and four-class load-bearing ratio levels, respectively, a comprehensive recognition accuracy of 94.3% for 28 fine-grained coupled working conditions, and a cross-subject generalization accuracy of 93.5%. Its overall performance is significantly superior to that of traditional machine learning algorithms.

It should be noted that all experiments in this paper were conducted at a fixed walking speed of 3 km/h, and the generalization performance of the proposed model under variable-speed walking conditions has not been explored yet. In future research, we will focus on investigating the variation characteristics of plantar pressure during variable-speed walking and systematically test the generalization ability of the model under such dynamic scenarios to further improve its adaptability to real-world gait patterns. In addition, the DAGBM model proposed in this paper has not yet been deployed in a lightweight form, which limits its practical application in wearable real-time monitoring devices. Therefore, subsequent work will focus on optimizing the model’s inference speed and reducing its computational complexity while ensuring the model’s prediction accuracy. This optimization will enable the model to be effectively integrated into wearable devices, realizing real-time and continuous monitoring of plantar pressure, and delivering valuable methodological references for gait-analysis research simulating ground-based gravity-adaptation training scenarios.

## Figures and Tables

**Table 1:** Hyper-parameter search ranges for the proposed DAGBM model.

Hyper-Parameter	Search Range
Number of boosting iterations	{50, 100, 200, 300, 400}
Maximum tree depth	{3, 4, 5, 6}
Learning rate	{0.01, 0.05, 0.1, 0.2}
MMD loss weight	{0.1, 0.5, 1.0, 2.0}
RBF kernel bandwidth	{0.1, 1, 10}

**Table 2:** Experimental parameter settings.

Factors	Level	Setting Basis
Body Weight Support	0%, 10%, 20%, 30%, 40%, 50%, 60%	Covers normal gravity, mild/moderate hypo-gravity and near-complete weightlessness scenarios, and is compatible with aerospace/rehabilitation application requirements.
Weight Bearing	0%, 10%, 20%, 30%	Covers no-load, mild/moderate/high intensity load conditions, while avoiding gait distortion and personal injury caused by loads exceeding 30% body weight (BW).

**Table 3:** Physical coordinates of sensor measuring points (cm).

No.	X	Y	No.	X	Y	No.	X	Y	No.	X	Y
1	0	0	13	1	−0.7	25	2.6	−0.7	37	3.7	0
2	0	1.7	14	1.1	1.4	26	2.5	1.4	38	3.7	1.7
3	0	3.5	15	1.1	3.4	27	2.5	3.4	39	3.7	3.4
4	0	5.4	16	1.1	5.2	28	2.5	5.2	40	3.7	5.4
5	0	7.5	17	1.1	7.5	29	2.5	7.5	41	3.7	7.5
6	0.2	9.5	18	1.2	9.5	30	2.4	9.5	42	3.6	9.5
7	−0.5	11.6	19	1.2	11.6	31	2.9	11.6	43	4.6	11.6
8	−0.8	13.5	20	0.9	13.5	32	2.8	13.5	44	4.5	13.5
9	−1.8	15.3	21	−0.2	15.4	33	2.2	15.3	45	4.2	15.2
10	−2	17.1	22	−0.1	17.3	34	1.3	19.8	46	3.7	17.1
11	−2.4	19.6	23	−0.5	20	35	1.8	17.2	47	3.1	19.7
12	−2.2	21.6	24	−0.7	22.5	36	0.7	22.5	48	2.1	22.3

**Table 4:** Recognition accuracy of single working condition levels using different algorithms (%).

Algorithms	BWS (7 Classes)	Weight Bearing (4 Classes)
SGBoost	88.2	89.5
Random Forest	90.7	91.3
SVM	89.4	90.1
XGBoost	93.5	94.2
DAGBM	96.8	97.4

**Table 5:** Performance comparison for 28-class coupled working condition recognition (%).

Algorithms	Accuracy	Precision	Recall	*F*_1_ Score
SGBoost	82.6	81.9	82.3	82.1
Random Forest	85.1	84.7	85.0	84.8
SVM	83.9	83.2	83.5	83.3
XGBoost	91.5	91.2	91.3	91.2
DAGBM	94.3	94.0	94.2	94.1

## Data Availability

Data are available upon request due to privacy. The data presented in this paper are available on request from the corresponding author. The data are not publicly available due to the project’s funding requirement.

## Supplementary Materials

The following supporting information can be downloaded at: https://www.mdpi.com/article/10.3390/bios16100585/s1.
