# Supplementary material for: Gait Feature Analysis and Recognition Under Coupled Weight Support and Bearing Conditions via a Domain-Adaptive Gradient Boosting Machine
Source: Biosensors (Basel). 2026 Oct 19;16(10):585. doi: 10.3390/bios16100585 (PMC13702098; doi:10.3390/bios16100585)
Supplement: Supplementary file 1 [file biosensors-16-00585-s001.zip › biosensors-4562294-supplementary.pdf]

Algorithm: Domain Adaptive Gradient Boosting Machine (DAGBM)

```
Input: Training data source domain X_s, label Y_s; Target-domain data X_t
        Maximum boosting iterations T
        Learning rate η
        MMD loss weight λ_MMD
        RBF kernel bandwidth σ
Output: Trained DAGBM model F(x)
Initialize: F0(x) = mean(Y_s)
for t ← 1 to T do
    # Compute negative gradients (residuals) on source domain
    r_i = - ∂L(Y_s, F_{t-1}(X_s)) / ∂F_{t-1}(X_s),    i = 1,2,…,N_s
    # Fit base decision tree h_t to residuals r_i on source domain
    h_t = DecisionTreeRegressor(max_depth = d).fit(X_s, r_i)
    # Compute MMD discrepancy between source-domain and target-domain feature distribution
    MMD_dist = MMD(X_s, X_t, σ)
    # Composite loss: prediction loss + domain-alignment MMD regularization
    # Composite_loss = Loss(Y_s, F_{t-1}+η·h_t(X_s)) + λ_MMD · MMD_dist
    # Update additive boosting model
    F_t(x) = F_{t-1}(x) + η · h_t(x)
end for
return F(x) = F_T(x)

Function MMD(X_1, X_2, σ):
    # RBF-kernel-based maximum mean discrepancy
    K_11 = RBF_kernel(X_1, X_1, σ)
    K_22 = RBF_kernel(X_2, X_2, σ)
    K_12 = RBF_kernel(X_1, X_2, σ)
    mmd = mean(K_11) + mean(K_22) − 2·mean(K_12)
    return mmd

Function RBF_kernel(a, b, σ):
    return exp( − ||a−b||^2 / (2·σ^2) )
```

In this research, cross-subject data split is adopted. The hyper-parameters including (T, d, η, λ, σ) are determined by Bayesian optimization within the search ranges given in Table X. The base learner is a decision tree. MMD serves as a regularization term to reduce distribution shift between source and target domains.
